# Advancements in engineered exosomes for wound repair: current research and future perspectives

**DOI:** 10.3389/fbioe.2023.1301362

**Published:** 2023-11-14

**Authors:** Hailian Ye, Feng Wang, Guangchao Xu, Feihong Shu, Kunwu Fan, Dali Wang

**Affiliations:** ^1^ Department of Burns and Plastic Surgery, Affiliated Hospital of Zunyi Medical University, Zunyi, Guizhou, China; ^2^ The Collaborative Innovation Center of Tissue Damage Repair and Regeneration Medicine of Zunyi Medical University, Zunyi, Guizhou, China; ^3^ Department of Burn and Plastic Surgery, Department of Wound Repair, Shenzhen Institute of Translational Medicine, The First Affiliated Hospital of Shenzhen University, Shenzhen Second People’s Hospital, Shenzhen, Guangdong, China

**Keywords:** exosomes, engineered exosomes, wound repair, precision therapye, biomaterials

## Abstract

Wound healing is a complex and prolonged process that remains a significant challenge in clinical practice. Exosomes, a type of nanoscale extracellular vesicles naturally secreted by cells, are endowed with numerous advantageous attributes, including superior biocompatibility, minimal toxicity, and non-specific immunogenicity. These properties render them an exceptionally promising candidate for bioengineering applications. Recent advances have illustrated the potential of exosome therapy in promoting tissue repair. To further augment their therapeutic efficacy, the concept of engineered exosomes has been proposed. These are designed and functionally modifiable exosomes that have been tailored on the attributes of natural exosomes. This comprehensive review delineates various strategies for exosome engineering, placing specific emphasis on studies exploring the application of engineered exosomes for precision therapy in wound healing. Furthermore, this review sheds light on strategies for integrating exosomes with biomaterials to enhance delivery effectiveness. The insights presented herein provide novel perspectives and lay a robust foundation for forthcoming research in the realm of cutaneous wound repair therapies.

## 1 Introduction

The skin, situated in the outermost layer of the human structure, holds the distinction of being the largest organ in the human body (Wang et al., 2023a; Yang et al., 2023a). It primarily serves as an immune defense and protective barrier against external pathogens and harmful substances while regulating the body’s metabolism and facilitating sensory perception. However, skin is susceptible to damage from various intrinsic pathologies and external mechanical factors, particularly burn wounds and chronic skin ulcers (Wang et al., 2022a; Chen et al., 2023; Osborne et al., 2023). Wound healing is a multifaceted process comprising four distinct and overlapping phases: haemostasis, inflammation, proliferation, and remodeling (Yang et al., 2023b). These processes encompass a range of intricate mechanisms, including the regulation of including the regulation of inflammation, angiogenesis, remodeling of the extracellular matrix (ECM), and cell proliferation, differentiation and migration (Nourian Dehkordi et al., 2019). The secretion of various cytokines, chemokines, and growth factors plays a crucial role in tightly controlling these processes. Any injury during this process can disrupt wound healing, leading to unsightly scar formation and affecting both aesthetics and functionality. Enhancing wound healing and minimizing scar formation remain formidable challenges in the field of wound repair treatment (Rani and Ritter, 2016).

In recent decades, significant research efforts have been focused on developing treatments that facilitate wound healing and inhibit scar formation. Traditional approaches have mainly centered on debridement of necrotic tissue, infection control, and the application of growth factors to promote granulation tissue formation, fostering a favorable microenvironment for wound healing (Fadilah et al., 2023; Lin et al., 2023; Ni et al., 2023). Nevertheless, current strategies for wound repair treatment have limitations, especially for severe burns and chronic skin ulcers, due to persistent inflammation, impaired angiogenesis, and inadequate extracellular matrix remodeling within the wound microenvironment (Liu et al., 2022a).

The recent advent of regenerative medicine provides a promising new direction in the field of wound repair. Stem cells, which serve as the origin of diverse mature cells in the human body, hold significant promise in the field of regenerative medicine owing to their remarkable immunomodulatory and regenerative capabilities (Hilton et al., 2019). Extensive studies have demonstrated the therapeutic potential of stem cells and their derived exosomes in addressing various conditions, including spinal cord injury (Ramalho et al., 2018), myocardial injury (Qiao et al., 2019), wound repair (Kou et al., 2018), bone defects (Zhang et al., 2021a), as well as inflammatory (Jun et al., 2023), fibrotic (Hu et al., 2022), and autoimmune diseases (Xu et al., 2022). It is evident that this strategy offers a wealth of promising therapeutic options for a wide array of medical conditions. In the realm of wound repair, stem cell transplantation and stem cell-derived exosomes into burns and diabetic chronic wounds have shown regenerative capacity by improving wound inflammation, promoting granulation tissue formation, and improving angiogenesis to facilitate healing of chronic diabetic wounds (Gadelkarim et al., 2018; Guillamat-Prats, 2021). Research has revealed that the use of mesenchymal stem cell (MSC) transplants or exosomes derived from stem cells can elicit distinct biological effects at different stages of wound healing (Choi et al., 2018). For instance, in the early stage of wound healing, transplantation of adipose MSCs into open wounds led to an upregulation of fibroblast collagen I and III expression, thereby promoting collagen synthesis and granulation tissue formation to accelerate wound healing (Bian et al., 2022). Conversely, in the late stage of wound healing, after the completion of epithelialization of wounds transplanted with adipose MSCs, their paracrine effect resulted in a downregulation of collagen synthesis to counteract fibrosis or scar formation (Li et al., 2021). However, the current application of MSCs and exosomes may overlook the scientific issue that when MSCs are transplanted as cells, their paracrine components and biological effects may vary depending on the disease-related pathological microenvironment. Therefore, the question arises as to how the microenvironment can be targeted to achieve specific therapeutic effects at different stages of wound healing. With a growing and deepening understanding of the skin regeneration process and wound healing, there is a burgeoning interest in precision treatment approaches for wounds (Su et al., 2019a; Vu et al., 2021).

As the research progresses, investigators have discovered potential key effector molecules within MSC-derived exosomes that can regulate the treatment of specific diseases (Baral et al., 2021; Rozier et al., 2021). Exosomes, small vesicles secreted by all cells, contain various substances such as nucleic acids and proteins, which play vital roles in intercellular communication. Moreover, exosomes are regarded as natural delivery vehicles due to their ability to penetrate tissues, high efficacy, long-term stability, ease of storage and transportation, controllable dosage and administration timing, and absence of tumor and thrombosis risks (Lelek and Zuba-Surma, 2020; Liang et al., 2021). Leveraging these inherent advantages, researchers have engineered exosomes as vectors to carry specific molecules required for disease treatment, thereby significantly enhancing their biological effects. Loading these key miRNAs into MSC-derived exosomes has emerged as a novel research direction to augment therapeutic efficacy (Beuzelin and Kaeffer, 2018; Zhixiao et al., 2021).

Furthermore, it has been observed that upon entering the circulation, exosomes tend to accumulate in organs such as the liver, lungs, and kidneys, with only a small fraction reaching the intended target site (Gangadaran et al., 2018). The targeting and efficacy of exosomal action largely depend on their interaction strength with targeted tissues and cells. By modifying the surface molecules of exosomes to confer cell- or tissue-specific targeting, their uptake by the desired tissue or cell can be enhanced. Such modifications enable precise targeting to the site of injury or the target cell (Antes et al., 2018; Kim et al., 2022). A growing body of research emphasizes the impact of exosomes on wound healing. Our proposed approach, involving engineered exosomes for cell-free therapy to facilitate precise wound treatment, holds immense promise. This strategy focuses on utilizing the key effectors necessary for the microenvironment during various stages of wound healing, leveraging the biological effects of exosomes. This review provides an overview of the advances of exosomes in the field of wound repair, describes the advantages and strategies of engineered exosomes, and discusses potential future research hotspots.

## 2 Exosomes in wound repair

### 2.1 Exosomes

All cells can release exosomes (Exos), which are vesicles that bud off the plasma mem-brane. The 2018 International Society for Extracellular Vesicles (ISEV) Guidelines (Théry et al., 2018) characterizes extracellular vesicles (EVs) as granules naturally secreted from the cell, encased in a lipid bilayer, and devoid of replication capability or a functional nucleus. These extracellular vesicles include exosomes, macrovesicles, microvesicles, microparticles, and apoptotic vesicles, with diameters ranging from 50 nm to 1 mm. Exosomes, in particular, have been extensively studied due to their distinct double lipid membrane structure and size, which measures approximately 40–160 nm in diameter, marking them as a subset of extracellular vesicles.

Nevertheless, to date, no reliable markers of subcellular origin have been established in experimental systems, and there is no consensus on specific markers for the complete isolation of extracellular vesicle (EV) subtypes. Following the MISEV2018 guidelines, researchers recommend adopting the terminological nomenclature of EV subtypes, replacing historically contradictory or biogenetically inaccurate terms such as exosomes and microvesicles (Théry et al., 2018). Importantly, the potential therapeutic applications of these subcellular components remain unaffected by the nomenclature of EVs or exosomes. In recent years, an increasing number of studies have classified them as EVs (Doyle and Wang, 2019).

**FIGURE 1:**
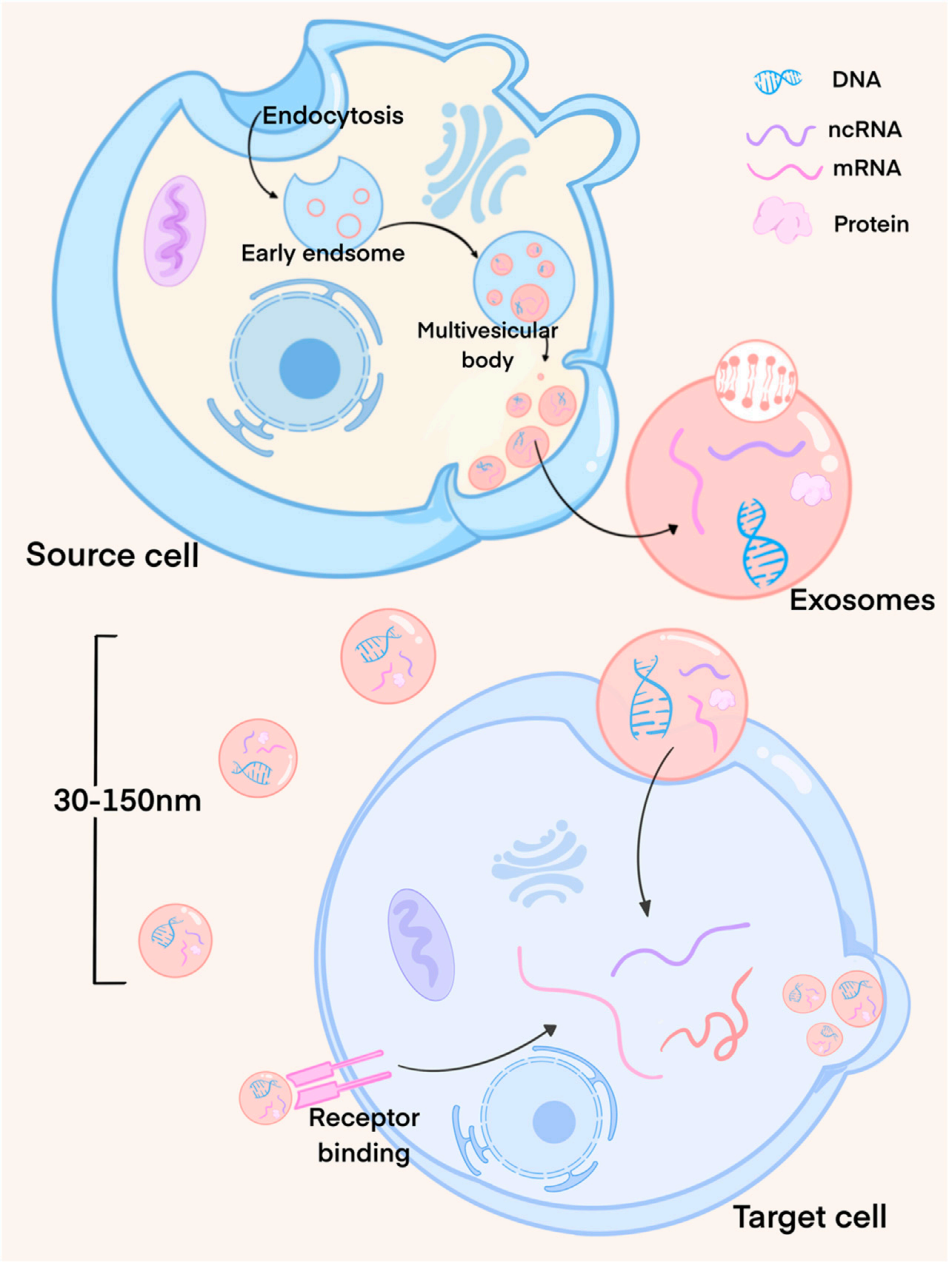
Exosome formation process. Exosomes mainly originate from the cells and bud inward from the plasma membrane to form multilayer intracellular bodies (MVBs). MVBs fuse with the plasma membrane to produce luminal vesicles (ILVs), which are then released as exosomes. At this time, exosomes load the cell’s mRNA, non-coding RNA, proteins and lipids through different mechanisms. Exosomes released by cells interact with recipient cells through endocytosis or membrane fusion.

Exosomes are unique in their biogenesis pathway, which is different from that of most other vesicles. They primarily originate within the cell, budding inward from the plasma membrane to form intracellular multilamellar bodies (MVBs). MVBs, upon fusion with the plasma membrane, generate intraluminal vesicles (ILVs) that are subsequently released as exosomes. The process of exosome biogenesis goes through several stages (Figure 1). During this process, a variety of substances, including intracellular mRNA, non-coding RNA, proteins, and lipids, are selectively loaded into the exosomes through various mechanisms and pathways, reflecting the biological properties of their parent cells. The exosomes are then directly secreted into the extra-cellular environment (Yang et al., 2023b).

Exosomes offer several advantages, including low toxicity and immunogenicity, along with a high transmembrane capacity, positioning them as key players in inter-cellular communication (Isaac et al., 2021). Once released from cells, exosomes interact with recipient cells through endocytosis or membrane fusion, delivering their cargo and thus inducing changes in the function and phenotype of the recipient cells. These changes, in turn, influence microenvironmental conditions and exert regulatory effects (van Niel et al., 2018; Kalluri and LeBleu, 2020; Yu et al., 2022).

It is noteworthy that exosomes exhibit a high degree of heterogeneity, leading to the activation of diverse pathways and induction of various biological functions (Jafari et al., 2019; Liang et al., 2021). This heterogeneity is influenced by factors such as size, content, and cell of origin (Zhang et al., 2021b). Differences in exosome size may result from the varying internal pressures generated during multivesicular body fusion with the plasma membrane and invagination, leading to differences in exosome content and composition (Kalluri and LeBleu, 2020). Jeppesen et al. (2019) reported that exosome morphology, particle size, and distribution vary depending on the cellular source, with tumor cell-derived exosomes being significantly larger. These findings were obtained through a refined method of exosome isolation. Similarly, Garcia-Martin’s study identified differences in protein composition among exosomes derived from various tissue sources through proteomic analysis (Garcia-Martin et al., 2022). For instance, exosomes from bone marrow mesenchymal stem cells (BMMSC) and adipose-derived stem cells (ADSC) differ significantly. Pomatto’s study investigated the biological activity of exosomes from both BMMSC and ADSC in promoting wound healing. Comparative analysis of the therapeutic properties of BMMSC-Exos and ADSC-Exos revealed that the molecules carried by ADSC-Exos are mainly related to angiogenesis, whereas the BMSC-Exos content is mainly associated with the proliferative process (Margherita et al., 2021). Due to these variations, exosomes originating from diverse sources may exhibit dissimilar biological effects in living organisms.

Exosomes can modulate recipient cells through two primary mechanisms. The first involves interactions between the membrane surfaces of exosomes and recipient cells. This interaction can occur through the binding of exosomal membrane surface proteins or biologically active lipid ligands to surface receptors on the target cells. Alternatively, exosomes can fuse with the cell membrane of recipient cells, facilitating the transfer of functional contents. The second mechanism involves the removal of exosomes from recipient cells through processes such as pinocytosis or endocytosis, allowing the internalized exosomal contents to influence the recipient cell’s function.

### 2.2 Exosomes and wound repair

Recently, there has been an increased emphasis on the role of exosomes in various physiological processes and their regulatory function under pathophysiological conditions. These versatile vesicles demonstrate the ability to regulate wound inflammatory responses, stimulate wound angiogenesis, and control extracellular matrix remodeling by transferring their functional cargo to target cells. As a promising cell-free therapeutic approach, exosomes have garnered significant attention for their potential in wound repair. Particularly, exosomes derived from stem cells have been found to possess similar biological properties and exhibit potent tissue repair and regenerative capabilities as their source cells (Than et al., 2017; Zhou et al., 2022).

For example, studies have shown that exosomes derived from mesenchymal stem cells (MSCs) can modulate the inflammatory response of wounds by promoting the polarization of macrophages towards the M2 phenotype and enhancing the secretion of antiinflammatory factors, thereby facilitating wound healing (He et al., 2019). Similarly, exosomes derived from human induced pluripotent stem cell-derived MSCs (hiPSC-MSCs-Exos) have been shown to promote angiogenesis, stimulate fibroblast proliferation, and boost collagen synthesis, all of which contribute to wound healing (Zhang et al., 2015). Despite functional differences among exosomes derived from different cell types, all appear to exert similar therapeutic effects on wound repair. Nonetheless, the clinical application of exosomes remains in its early stages, with various challenges that must be addressed before they can be widely adopted. These challenges encompass issues such as low yield, time-consuming and costly production processes, and quality control concerns that necessitate extensive clinical validation (Wa et al., 2022; Kim et al., 2023).

## 3 Engineered exosomes

Recent studies increasingly show that exosomes, obtained through pretreatment (e.g., cytokines, hypoxia, 3D culture, various physical or chemical stimuli) or engineering strategies, display more specific biological activities and yield better therapeutic effects compared to exosomes derived solely from MSCs (Miceli et al., 2021; Chen et al., 2022a). For instance, Wang et al. (2021) showed that exosomes derived from ADSCs obtained after hypoxic pretreatment exhibit enhanced fibroblast proliferation and migration through the PI3K/AKT signaling pathway, and contribute to improved healing outcomes in chronic wounds, exceeding the capabilities of exosomes derived under normoxic conditions. The pre-treatment strategy significantly altered the components of the MSC secretome and enhanced the biological effects of MSC-derived exosomes. However, the factors responsible for the observed biological differences remain unclear. Furthermore, researchers have proposed the modification of source cells or exosomes themselves to confer specific biological functions tailored to the requirements of wound healing. This approach enables precise regulation of the localization of different cells during the wound healing process, ultimately achieving the desired therapeutic outcomes (Yang et al., 2023b). Meng’s review describes several miRNA types associated with wound healing, and engineering effector miRNAs into source cells or exosomes can yield high-miRNA exosomes to enhance biological effects (Meng et al., 2018). Moreover, some studies focus on enhancing the therapeutic effect of exosomes by directly modifying them or by modifying the source cells to obtain them. Using a genetic engineering technique to introduce bone morphogenetic protein 2 gene (BMP-2) into BMMSCs, Li et al. (2022a) found that exosomes produced by engineered BMMSC led to enhanced bone regeneration. The BMP2 gene itself has an osteogenic role, and its combination with BMMSCs may act synergistically (Li et al., 2022a). Engineered modifications of exosomes have thus gained significant attention and importance in regenerative medicine, and stand as a key research focus in exploring exosome applications across various medical disciplines. Encouraging progress has been made in utilizing exosomes as drug delivery carriers, gene therapy vehicles, and in the realm of tumor therapy (Zhang et al., 2021b).

The exploration of engineered exosomes with enhanced targeting and effector properties for wound treatment is a topic that demands thorough examination. Modifying exosomes through genetic engineering to regulate their production, release, and intercellular communication holds great potential for enhancing their efficiency and efficacy in various applications. This approach involves genetic modifications of cells, exosome precursors, and exosomes themselves, allowing for the expression of functional molecules on the exosome membrane or their encapsulation within the exosome. By engineering exosomes, the limitations associated with current exosome applications can be effectively overcome. Engineered exosomes not only retain the desirable characteristics of natural exosomes, such as good biocompatibility, low immunogenicity, and cellular communication abilities, but also offer enhanced precision and advantages in terms of specific biological activities and therapeutic targeting. Consequently, they significantly improve the specificity, efficacy, and safety of exosome-based treatments, thereby demonstrating high clinical application value (Jafari et al., 2020; Hertel et al., 2022; Mondal et al., 2023).

**FIGURE 2:**
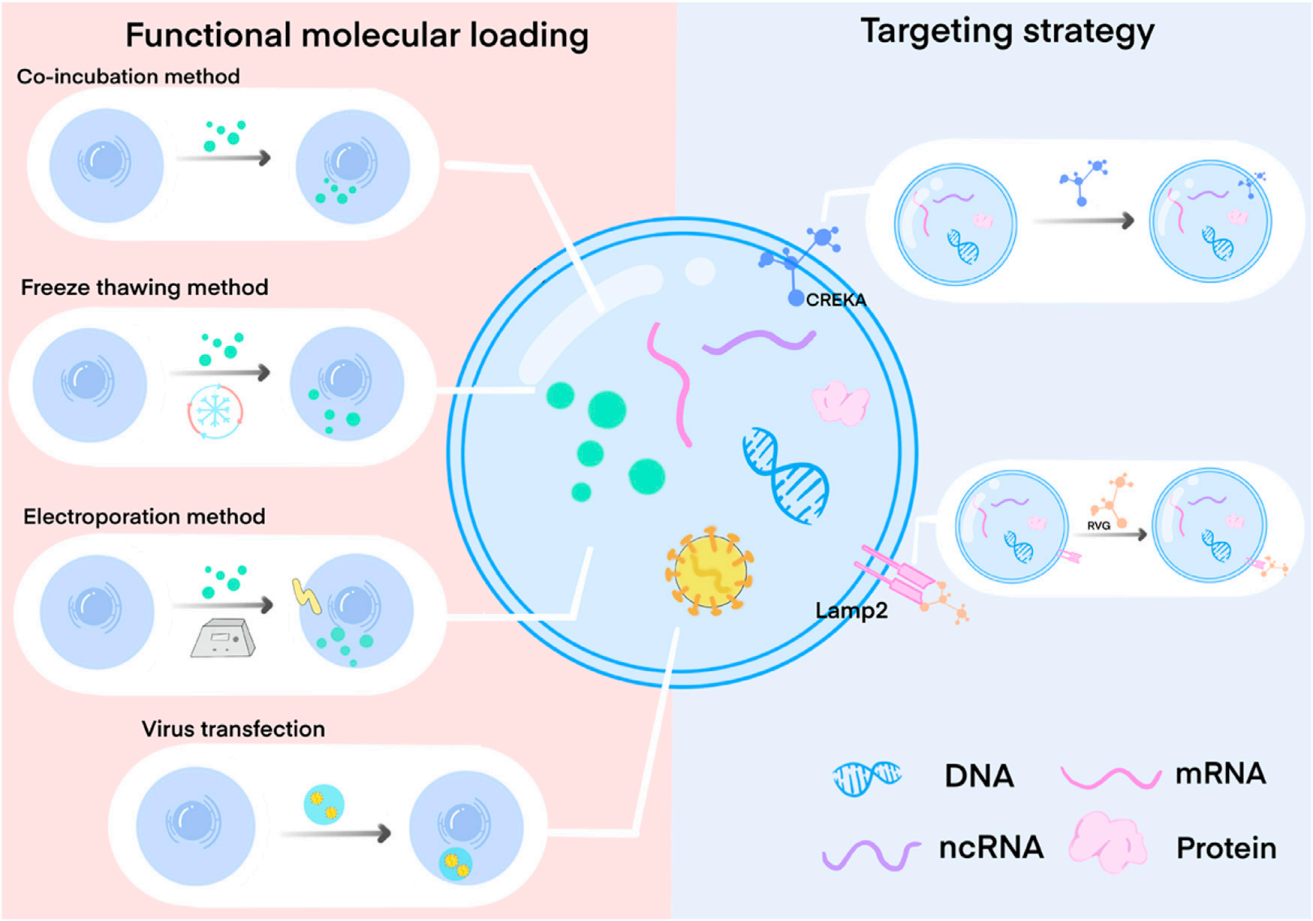
Exosome engineering method by loading functional molecules and surface targeted modification. Functional molecular loading mainly involves the modification of cells, exosome precursors, and exosomes through co-incubation, freeze-thaw, electroporation, virus transfection, etc. So that functional molecules are expressed on the surface of the exosome membrane or loaded into the exosome, thereby affecting the production and composition of exosomes. Targeted modification is mainly through the specific modification of exosome membrane surface proteins to specifically bind to the target cell surface receptors.

In this section, we present a succinct overview of bioengineering strategies used to modify exosomes, including improvements in bioactivity, enhanced targeting capabilities, and alterations in exosome loading molecules (Figure 2). We also summarize the research on the ap-plication of bioengineered exosomes with unique biological effects in wound repair therapy, focusing on critical factors involved in the wound healing process. We will further discuss strategies for exosome delivery through biomaterial binding. Lastly, we will discuss the advancements and prospects of engineered exosomes within the field.

### 3.1 Functional molecular loading

Exosomes, which are derived from cells, can be modified by manipulating source cells to acquire specific functionalities (Liang et al., 2021; Ferreira et al., 2022). The most common approach involves loading functional molecules into cells, which influences exosome production and composition. Various techniques are used for cell modification, including co-incubation, membrane permeation, electroporation, transfection, ultrasound methods, extrusion methods, and freeze-thawing (Liao et al., 2019; Liang et al., 2021; Ferreira et al., 2022). Additionally, functional molecule loading techniques can be employed to directly load therapeutic molecules into exosomes, thereby obtaining exosomes with specific therapeutic properties (Liang et al., 2021; Ferreira et al., 2022).

The co-incubation method is a commonly employed technique in studies involving exosome drug loading, known for its simplicity. This method primarily relies on the principle of simple diffusion, where the therapeutic drug is co-incubated with exosomes or source cells for a specific duration, allowing the drug to diffuse into the exosomes or their source cells along a concentration gradient. This diffusion process primarily occurs through the interaction between the drug’s hydrophobicity and the lipid layer of the membrane (Luan et al., 2017; Xi et al., 2021). For instance, Curcumin, a hydrophobic substance with natural anti-inflammatory activity but unstable in nature, can self-assemble into the lipid bilayer of exosomes through hydrophobic interactions between hydrophobic tails and hydrophobic drugs. Sun et al. (2010) successfully obtained curcumin-containing exosomes using the co-incubation method, curcumin solubility and stability *in vitro* are improved by combining curcumin with exosomes, leading to enhanced anti-inflammatory activity and bioavailability of curcumin. However, it is important to note that the co-incubation method often suffers from inefficiency and limited success in drug loading due to the absence of factors facilitating drug diffusion and specificity.

Haney et al. (2015) conducted a comparative analysis of various processing methods for exosomes, including co-incubation, freeze-thaw, sonication and extrusion (Zhu et al., 2021a). Their findings revealed that sonication and extrusion exhibited the highest loading efficiency and activity. They concluded that these methods caused the exosome membrane to become more permeable, allowing drug diffusion and loading into the exosomes. The freeze-thaw method capitalizes on the changes in membrane permeability that occur during repeated freezing and thawing to facilitate drug or molecule diffusion into the exosomes (Ma et al., 2023). Won Lee et al. (2020) demonstrated the effective loading of miR-140 into nanoscale EVs derived from plasma through isolation and ten repeated freeze-thaw cycles. These EVs, known as miR-140-Exo, possess the bioactive function of miR-140, which is crucial for cartilage tissue stability. The loaded miR-140-Exo was successfully utilized to promote the differentiation of BMMSCs into chondrocytes. Notably, no structural changes were observed in miR-140-Exo during the loading process (Won Lee et al., 2020). Although the physical methods mentioned above are simple and cost-effective, they may induce alterations in the structure of the exosome membrane, potentially impacting its morphology and integrity, consequently diminishing effective membrane binding.

Studies have indicated that ultrasound stimulation can increase the size of exosomes, potentially due to drug loading. However, the morphology of the exosomes and the surface labeling proteins remained unchanged. Furthermore, ultrasound stimulation did not appear to affect the protein binding of exosome membranes, thereby demonstrating the suitability of this method for loading drugs into exosomes (Wang et al., 2019a). Ultrasound engineering has been widely reported in the literature for modifying exosomes used in oncology drug delivery systems. The process involves mixing drug and nanocapsule fillings in appropriate proportions, followed by sonication using a Model 505 Ultrasonic Disintegrator equipped with a 0.25″ needle. The sonication conditions include a 20% amplitude and 6 cycles of 30 s on/off for a total duration of 3 min, with 2 min of cooling between cycles. The mixture should be incubated at 37°C for 1 h, or alternatively, the medication and nanovesicles can be mixed in equal quantities, stirred well, and incubated at room temperature (RT) for 1 h (Wan et al., 2018). Wang et al. (2019a) demonstrated that loading paclitaxel (PTX) into M1 macrophages using a mild ultrasound method resulted in the isolation of M1-Exos, which increased caspase-3 activity in cancer cells through the pro-inflammatory effect produced by M1-Exos and enhanced anti-tumor activity via a caspase-3 mediated pathway. Similarly, Chen et al. (2022a) loaded rifampicin into exosomes derived from BMSCs using ultrasound. *In vitro* experiments showed that this process could inhibit the growth of osteosarcoma (OS) cell lines with low toxicity. Compared to the control group, the treated OS mice had a longer survival period, indicating the potent anti-tumor therapeutic effect of EXO-RIF. Additionally, the study found that EXO-RIF activated dynamically related protein 1 (Drp1) and induced mitochondrial apoptosis (Chen et al., 2022a). However, it is important to consider that ultrasonic stimulation may mimic electroporation, as both methods can facilitate the delivery of molecules across membranes. Further research is needed to fully understand the mechanisms and effectiveness of ultrasonic stimulation compared to electroporation. It is also crucial to consider the potential biological effects and safety implications of using either method for drug delivery or other applications. Nonetheless, the possibility of using ultrasonic stimulation as a promising approach for loading drugs into exosomes cannot be ruled out.

Electroporation, a relatively straightforward method, involves creating pores in the exosome membrane using high voltage, allowing for the loading of external target molecules near the endosomal cavity (Gilligan and Dwyer, 2017). Xiong et al. (2023) reported that miR-542-3p targeted stimulation of human skin fibroblasts (HSFs)/human dermal microvascular endothelial cells (HMECs), and successfully modified exosomes to target the wound healing mechanism by introducing miR-542-3p into bone marrow mesenchymal stem cells (BMSCs) by electroporation. This modification enhanced the exosome’s pro-angiogenic and pro-cell proliferative effects, enabling effective wound treatment (Yan et al., 2022). However, it is important to note that this simple electroporation approach still presents certain limitations, such as potential exosome cleavage, aggregation, and partial membrane incompleteness (Lamichhane et al., 2015). To overcome this limitation, Hood et al. (2014) developed a novel trehalose pulsed medium (TPM) consisting of 50 mM trehalose in PBS. The use of TPM minimized exosome aggregation after electroporation compared to exosomes in PBS, particularly at electric fields of 0.75 and 1.5 kV/cm. Notably, a 0.75 kV/cm electric field uniquely dispersed exosomes. Additionally, TPM demonstrated its advantage in preserving the stability of exosomes during cold storage. This finding was consistent for both melanin-derived exosomes and exosomes obtained from human serum (Hood et al., 2014). Moreover, in laboratory settings, electroporation requires a large quantity of exosomes to enhance the yield (Hood et al., 2014).

Furthermore, viral transfection can also be employed to modify source cells. This involves inserting relevant DNA fragments into the genome of source cells, subsequently collecting exosomes secreted by these genetically modified cells that possess ligands (Wan et al., 2018). For example, McAndrews et al. reported that CRISPR/Cas9, a gene editing system, was used to target the KrasG12D gene, which suppresses pancreatic cancer, and was packaged as a plasmid DNA and delivered to the recipient cancer cells through exosomal vector by transfection to induce the deletion of the target gene for precision targeting therapy (McAndrews et al., 2021). However, to enhance the therapeutic effects of exosomes, transfection is often used to transfer molecules with critical therapeutic effects (such as proteins and miRNAs) into source cells or exosomes (Liao et al., 2019). Sun et al. (2023) reported the successful application of engineered exosomes through BMP2 proteins with bone-forming effects transfection in mouse embryonic fibroblast (NIH-3T3) cells were loaded on GelMA hydrogels for tissue regeneration. Additionally, miR-132 has been identified as a crucial microRNA for wound healing, possessing anti-inflammatory capacity and pro-angiogenic effects (Li et al., 2015; Li et al., 2017). Lentiviral transfection was used to obtain adipose-derived stem cells (ADSC) overexpressing miR-132, and their exosomes demon-strated significant efficacy in promoting wound healing (Ge et al., 2023).

However, experimental studies that use transfection methods to engineer exosomes have demonstrated a lower efficiency in achieving successful transformation, making it challenging to obtain sufficient quantities of exosomes successfully loaded with therapeutic molecules for subsequent application studies (Li et al., 2022a).

### 3.2 Targeted therapy

Upon intravenous administration, it has been observed that MSC-derived exosomes accumulate primarily in the liver, lungs, and spleen, while the concentration of exosomes reaching the disease’s target tissue remains low. This results in diminished effective communication between exosomes and recipient cells, posing challenges in achieving desired therapeutic outcomes (Grange et al., 2014; Lai et al., 2014; Schmuck et al., 2016). This distribution of exosomes was also observed in the veins of rats and mice using exosome injections labeled with zirconium-89 (89Zr), as demonstrated by Choi et al. (2022) Furthermore, it was demonstrated that cells and tissues may rapidly take up exosomes penetrating the body. Therefore, the biological effects of exosomes can be effectively promoted by developing a strategy that enables targeted and precise delivery of exosomes to damaged sites (Liu et al., 2020a). To address this, researchers have pro-posed surface modifications of exosomes by adding targeted proteins to their surface or fusing them with other molecules. These modifications enhance the expression of specific proteins on the exosome surface that can readily bind to and engage recipient cells. Moreover, these modifications enable targeted binding to recipient cells or uptake by recipient cells, thereby facilitating the regulation of recipient cell function. For instance, Nakase reported that CPP, an arginine-rich cell-penetrating peptide modified on the exosome membrane, activated the phagocytic pathway of large protein cells, resulting in a significant enhancement of exosome uptake. To clarify, this suggests that the use of CPP led to an increase in the absorption of exosomes by the cells (Nakase et al., 2017). Engineering strategies like these greatly enhance the targeted and highly effective therapeutic out-comes of engineered exosomes. When targeted exosome engineering strategies are employed, the likelihood that exosomes reach desired cells or tissues during treatment is significantly increased. This, in turn, amplifies the targeting precision and efficiency of engineered exosomes, leading to synergistic therapeutic effects (Jafari et al., 2020; Li et al., 2022b).

Currently, targeted fusion utilizing exosomal signal peptides stands as the most widely employed method for achieving targeted modification (Salunkhe et al., 2020). For instance, rabies virus glycoprotein (RVG) targeting peptides exhibit neural specificity, making them a popular choice for targeting purposes. By fusing the RVG targeting peptide with the exosomal membrane protein Lamp2 and introducing this modified construct into dendritic cells, engineered exosomes with neurospecific targeting capabilities can be secreted. These engineered exosomes serve as carriers for loading exogenous siRNA, enabling specific delivery to therapeutic targets in Alzheimer’s disease for therapeutic interventions (Alvarez-Erviti et al., 2011; Salunkhe et al., 2020). Notably, the RVG-targeted peptide modification results in approximately twice the brain targeting efficiency compared to the unmodified group, showcasing high specificity and effective enrichment at the therapeutic site (Wiklander et al., 2015). These studies underscore the feasibility of exosomal membrane surface targeting modifications. However, it is crucial to care-fully consider the selection of exosomal modified membrane proteins during application (Richter et al., 2021). Additionally, the utilization of signal peptides for targeting fusion to exosomal membrane proteins may potentially impact the normal function of these proteins (Salunkhe et al., 2020).

In addition, certain researchers have explored target modification through bio-coupling target ligands with exosomal membrane surface proteins or by enhancing ligand-receptor interactions (Salunkhe et al., 2020; Liang et al., 2021). Studies have demonstrated that coupling tumor cell-derived exosomes with two distinct ligands (biotin and affin) enhances their active targeting capability, rendering them more effective carriers for drug delivery and im-proving the anticancer efficacy (Wang et al., 2017). Furthermore, the hydrophobic insertion method was employed to modify exosomes with the fibronectin-targeting peptide CREKA, facilitating its ability to target fibronectin and enrich it at sites of tissue defects (Qi et al., 2023). Results show that exosomes modified using this surface modification approach demonstrate specific targeting and effectively promote tissue repair without compromising the exosomes’ original biological activity. Nevertheless, modifying the membrane surface of exosomes could potentially lower the efficiency of communication between exosomes and recipient cells, or even impact exosome structure and function. This approach could also inactivate membrane surface proteins or cause exosome aggregation, necessitating further investigation and development.

Recently, Zhu et al. (2022) reported the construction of a dual-targeted functionalized exosome containing Angiopep-2 and TAT and loaded with therapeutic drugs, which can target the therapeutic site and increase the concentration of therapeutic drugs. The researchers fused angiogenic peptide and TAT peptide into Ang-Lamp2b-HA and TAT-Lamp2b-EGFP fragments, respectively, which were then inserted into lentiviral vectors to transfect HEK293T cells. New cell lines were obtained, which stably expressed fusion proteins of Ang-Lamp2b-HA and TAT-EGFP-Lamp2b. Their exosomes were isolated, purified, and then mixed with Dox. Thereafter, the drug was loaded into the exosomes by electroporation at 350 V, followed by extraction of exosomes from the cells and addition of the drug. The Angiopep-2 peptide targets low-density lipoprotein receptor (LRP-1), whereas the transmembrane peptide TAT enhances exosome permeability across the blood-brain barrier and tumor tissues (Zhu et al., 2022).

### 3.3 Engineered exosomes and wound repair

Wound healing is a multifaceted process encompassing coagulation, reepithelialization, granulation tissue formation, and revascularization (Lu et al., 2022). It can also be categorized into four interconnected phases: hemostasis, inflammation, proliferation, and remodeling. Recent studies have highlighted the involvement of exosome therapy in all phases of wound healing, actively promoting the healing process. For instance, exosomes regulate inflammation, facilitate macrophage polarization towards the M2 phenotype, promote angiogenesis, regulate cell migration, proliferation, and collagen synthesis, and modulate remodeling to minimize scar formation (Yang et al., 2015; Hu et al., 2016; Kang et al., 2016; Yang et al., 2023c). Despite promising results from research on the regenerative applications of exosomes in skin trauma, their clinical translation efficacy falls short of meeting clinical demands. Previous studies have identified specific molecules within exosomes that contribute to wound healing. Consequently, researchers have employed various engineering techniques to augment the levels of these components in cells or exosomes to better align with the physiological processes of wound healing, enabling the generation of enhanced exosomes with specific functions (Table 1). This approach allows precise regulation of the local-ization of different cells during the wound healing process. In the following sections, we present relevant research related to the application of engineered exosomes in control-ling inflammation and promoting angiogenesis during wound healing.

**TABLE 1:** Summary of research investigating engineered exosomes involvement in wound healing.

Cargo	Aim	Exos-souces	Engineering methodology	Wound type	Pathways; effector cell	Function	References
Poildopamine (PDA)	Antioxidant properties aid in anti-inflammation	milk	Co-extrusion	Skin wound	PI3K-AKT pathway; Promotes the growth, migration and anti-inflammatory responses of 3T3 cells	Anti-inflammatory, antiangiogenic, and collagen stimulating	Fan et al. (2023)
Dapagliflozin (DA)	Sodium-glucose linked transporter 2 (SGLT2) inhibitor	Induced pliripotent stem cells-derived Endothelial cells (ECs)	Extrusion	Diabetic wounds	HIF-1α/VEGFA pathway	Angiogenesis enhancement	Zhang et al. (2023)
Transcripts [ETV2, FLI1, and FOXC2 (EFF)]	Reprogram somatic cells to iECs	Human Dermal Fibroblasts (HDFs)	Electroporation	Skin wound	induced Endothelial Cells (iECs)	Promote angiogenesis	Rincon-Be et al. (2023)
miR-542-3p	—	Bone marrow Mesenchymal stem cells (BMSC)	Electroporation	Skin wound	Human Skin Fibroblasts (HSFs)/Human dermal Microvascular Endothelial Cells (HMECs)	Cell proliferation, Collagen deposition, Neovascularization, and Accelerated wound healing	Xiong et al. (2023)
miR-31-5p	Promote angiogenesis	Milk	Electroporation	Diabetic wounds	Endothelial cells	Promote angiogenesis	Yan et al. (2022)
miR-21-5p	—	Adipose Stem Cells (ADSC)	Electroporation	Diabetic wounds	Wnt/β-catenin signaling; Keratinocytes	Re-epithelialization, Collagen remodeling, Angiogenesis	Lv et al. (2020)
miR-132	—	miR-132-adipose stem cells (miR-132-ADSC)	Viral transfection	Diabetic wounds	NF-κB pathway; Human Umbilical Vein Endothelial Cells (HUVECs)	Reduce local inflammation, promote angiogenesis, and stimulate M2 macrophage polarization	Ge et al. (2023)
miR-126-3p	—	Adipose Stem Cell (ADSC)	Transfection	Skin wound	PIK3R2; Fibroblasts/Human Umbilical Vein Endothelial Cells (HUVECs)	Fibrocyte proliferation, Promote angiogenesis	Ma et al. (2022a)
miR -126-3p	Promote angiogenesis	Synovium Mesenchymal Stem Cells (SMSCs)	Viral transfection	Diabetic wounds	Human dermal Fibroblasts and Human dermal Microvascular Endothelial Cells (HMEC-1)	Fibrocyte proliferation, Promote angiogenesis	Tao et al. (2017)
Tumor necrosis factor (TNF)-stimulated gene-6 (TSG-6)	anti-inflammatory	Human bone marrow mesenchymal stromal cells (hBMSCs)	Viral transfection	Skin wound of mices	—	Reduction of inflammation and collagen deposition	Jiang et al. (2020)
HOX transcript antisense RNA (HOTAIR)	Promote angiogenesis	Mesenchymal Stem Cells (MSCs)	Viral transfection	Diabetic Wounds	upregulation of the angiogenic protein vascular endothelial growth factor	Promote angiogenesis	Born et al. (2022)
*Nocardia* rubra cell wall skeleton (Nr-CWS)	Enhancement of macrophage activation and new blood vessel formation	Human Umbilical Cord Mesenchyreal Stem Cells (HUC-MSCs)	Preconditioning	Diabetic Wounds	circIARS1/miR-4782-5p/VEGFA axis; Human Umbilical Vein Endothelial Cells (HUVECs)	Promote angiogenesis	Li et al. (2023b)
Melatonin	—	Human bone marrow mesenchymals cells (hBMSCs)	Preconditioning	Diabetic wounds	PTEN/AKT pathway; M1 and M2 Macrophage	Inhibit inflammatory response	Liu et al. (2020b)
Metabolic Glycoengineering (MGE)	Adjust cellular metabolism to modulate glycosylation	MGE-Adipose Stem Cell (ADSC)	MGE-mediated Click chemistry	Inflammation	M1 and M2 Macrophage	Inhibit inflammatory response	You et al. (2021)
IRF1	Fibrocyte proliferation, Promote angiogenesis	Rat Adipose-derived stem cell (ASC)	Viral transfection	Diabetic wounds	Regulates miR-16-5p/SP5 axis	Promotes wound healing	Wu et al. (2023)
Blue Light	Promote angiogenesis	Human Umbilical Cord Mesenchyreal Stem Cells (HUC-MSCs)	Preconditioning	Skin wound of mices	MiR-135b-5p and miR-499a-3p were upregulated; Human Umbilical Vein Endothelial Cells (HUVECs)	Promote angiogenesis	Yang et al. (2019)
Hypoxic	—	Adipose-derived stem cells (ADSCs)	Preconditioning	Diabetic Wounds	miR-144–3p/HIF-1α axis; Induces polarization of M2 macrophages	Promotes wound healing	Shi et al. (2022)
Selenium	Enhance the proliferation, multipotency, and anti-inflammatory effects of mesenchymal stem cells (MSCs)	Adipose Stem-Cell (ADSC)	Preconditioning	Skin wound of mices	Upregulation of remodeling factors; Contains miRNAs beneficial for healing wounds	Selenium-Treated Exosomes Contained Many Beneficial miRNAs for Wound Healin	Heo (2022)
3,3′-diindolylmethane (DIM)	regulate the key signal pathway	Human Umbilical Cord Mesenchyreal Stem Cells (HUC-MSCs)	Preconditioning	Burn injury rat model	Wnt/β-catenin signaling	Huc-MSC stemness enhancement	Hui et al. (2017)
miR-503	Target to IGF1R	M1 macrophage	Viral transfection	Diabetic Wounds	miR-503/IGF1R axis; Human umbilical vein endothelial cells (HUVECs)	M1-macrophage-induced HUVEC dysfunction was attenuated by miR-503 inhibition in HG-stimulated macrophages	Wang et al. (2023b)
Lamp2b	Target to Cardiomyocyte Specific Peptide (CMP)	Cardiosphere - derived Cells (CDCs)	Viral transfection	—	—	Improve uptake of exosomes by target cells	Mentkowski and Lang (2019)
LTH	Target to kidney injury molecule-1 (Kim-1)	Blood cell	Coupled reaction	—	—	Improve uptake of exosomes by target cells	Tang et al. (2021)

#### 3.3.1 Regulation of inflammation and oxidative stress

Inflammation is a complex regulatory process that serves as the body’s response to tissue injury or infection. Maintaining a balanced population of inflammatory cells within the regenerative microenvironment is crucial for successful wound healing (Zhu et al., 2021b; Meng et al., 2021; Wang et al., 2022b). In the case of chronic wounds, the inflammatory state and oxidative stress are more severe compared to normal wounds. This heightened inflammation and oxidative stress contribute to cellular damage and death, resulting in tissue dysfunction and impaired wound healing (Long et al., 2016; Han and Ceilley, 2017).

Studies have demonstrated the ability of exosomes to mitigate the inflammatory response in the treatment of chronic wounds (Li and Wu, 2022). These effects are achieved through the reduction of oxidative stress, modulation of inflammatory factor secretion, and regulation of macrophage polarization. Furthermore, engineered exosomes exhibit superior therapeutic efficacy due to their inherent advantages in inflammation regulation com-pared to natural exosomes. Engineered exosomes possess higher concentrations of therapeutic molecules, enhanced targeting abilities, and improved stability, while retaining the essential properties of exosomes. This enables the incorporation of specific functional molecules to enhance therapeutic capabilities. Consequently, these engineered exosomes not only address the issues of low yield and efficiency associated with natural exosomes but also facilitate the implementation of diverse and combined therapeutic strategies for inflammatory diseases (Ma et al., 2023).

Furthermore, studies have demonstrated that exosomes carrying miRNAs involved in inflammatory regulation can promote wound healing through a synergistic effect, inhibiting oxidative damage and tissue inflammation. These engineered exosomes exhibit superior therapeutic effects compared to natural exosomes alone. Additionally, surface-targeted modification of exosomes derived from engineered stem cells has been shown to achieve more efficient anti-inflammatory effects by specifically targeting the sites of inflammation (You et al., 2021).

Moreover, studies have reported that engineered exosomes can suppress inflammatory responses. For instance, lentiviral transfection can be utilized to obtain exosomes overexpressing PD-1, which negatively regulates T-cell activity by binding to inhibitory PD-1 receptors on T cells. This approach effectively suppresses inflammatory cytokine production and promotes wound healing (Su et al., 2019b).

It is important to note that the effect of exosomes on the inflammatory response is influenced by the cell type from which they originate and the specific interventions or conditions within the source cell culture environment. For instance, exosomes derived from stem cells exhibit an inhibitory effect on the inflammatory response of recipient cells, thereby reducing overall inflammation. Conversely, exosomes derived from immune cells can activate the inflammatory response, leading to increased inflammation and inflammatory diseases.

Therefore, when designing engineered exosomes, it is crucial to consider whether to harness their synergistic anti-inflammatory effect or utilize their potential to modulate the immune response in a reverse manner. However, regardless of whether exosomes exhibit inhibitory or activating effects on the inflammatory response, it is primarily mediated through the microRNA content present within the exosomes (Noonin and Thongboonkerd, 2021).

#### 3.3.2 Angiogenesis

Angiogenesis plays a vital role in the wound healing process, as it is responsible for maintaining nutrient transport, oxygen homeostasis, and contributing to wound healing and tissue regeneration. This complex process involves vascular endothelial cells and various angiogenesis-related factors (Rodrigues et al., 2019; Li and Wu, 2022). Notably, exosomes have been identified as effective promoters of endothelial cell proliferation, migration, and angiogenesis through multiple pathways, significantly enhancing local vascular regeneration on traumatized surfaces (Dong et al., 2023).

Several studies have successfully loaded specific miRNAs that promote angio-genesis into exosomes using techniques like electroporation or viral transfection. For instance, engineered exosomes containing overexpressed miR-126 were prepared through lentiviral vector transfection and applied to diabetic wounds, resulting in accelerated re-epithelialization, angiogenesis, and improved wound healing (Tao et al., 2017). In addition, electroporation techniques have shown promise in transferring miR-31-5p into exosomes derived from milk (Lv et al., 2020). *In vitro* experiments revealed that the application of engineered milk-derived exosomes facilitated cellular uptake and enhanced endothelial cell function, leading to significant promotion of angiogenesis and facilitation of the wound healing process. Similarly, the introduction of miR-21-5p mimics into exosomes derived from human adipose mesenchymal stem cells through electroporation has shown promising results in promoting re-epithelialization, collagen remodeling, angiogenesis, and expediting the healing of diabetic wounds (Ma et al., 2022a).

Moreover, some studies have explored the direct encapsulation of exosomes with VEGF plasmid DNA to augment vascularization (Zha et al., 2020). These findings highlight the potential of loading exosomes with miRNAs that possess significant regenerative effects, offering a strategic foundation for drug delivery and cell-free therapies (Lou et al., 2022).

Furthermore, it has been observed that alterations in the cell culture environment can impact the functionality of exosomes. Although this approach does not specifically target the modification or loading of functional molecules to modify cells, it does en-hance exosomes by modifying their composition (Dong et al., 2023). For instance, when comparing exosomes derived from mesenchymal stem cells (MSCs) produced under normoxic and hypoxic conditions, no significant differences were observed in terms of yield, size, or surface characteristics (Almeria et al., 2019). However, exosomes produced in a hypoxic environment exhibited an augmented capacity to promote angiogenesis. Additionally, the application of 455 nm blue light was found to enhance the potential of MSC-derived exosomes in promoting endothelial cell and vascular regeneration (Yang et al., 2019).

However, the process of vessel formation is regulated by factors that promote or inhibit angiogenesis. When a wound is being repaired, it is necessary to reduce angiogenesis in order for proper physiologic wound healing to occur. In the early stages of wound healing, it is preferable for the wound environment to be pro-angiogenic to facilitate wound healing. Later, during the remodeling stage, it is more desirable for the wound environment to be less vascularized to reduce scar formation (Sharma et al., 2021; Rai et al., 2022).

Overall, various engineering methodologies to modify exosomes result in distinct exosomal effects. However, it is important to note that not all exosomes derived from various cells are suitable for engineering. As research on the mechanisms of wound healing and exosome biogenesis progresses, we can anticipate the emergence of more precisely engineered exosomes that regulate wound healing in a highly targeted man-ner (Lou et al., 2022).

#### 3.3.3 Regulation of the wound microenvironment

The wound regeneration microenvironment plays a pivotal role in the process of wound repair and exerts direct influence on several biological processes involved in wound healing. It encompasses both the external environment surrounding the wound and the internal environment within the wound tissue, which comprises cells, extra-cellular matrix, and extracellular tissue fluid (Scalise et al., 2015; Cheng and Fu, 2020). An unfavorable wound microenvironment can adversely affect treatment efficacy, making it challenging to achieve de-sired outcomes, particularly in the case of diabetic chronic wounds (Wang et al., 2022a). The detrimental effect of the wound microenvironment presents a significant hurdle in wound healing. Fortunately, engineered exosomes offer a promising solution to this predicament. For instance, Huang et al. (2021) successfully engineered exosomes loaded with miR-31 and demonstrated their ability to promote angiogenesis, as well as fibroblast and epithelial cell proliferation within the microenvironment of diabetic wounds, thereby enhancing wound healing. Furthermore, June suggests that selenium improves the secretion of exosomes by various cells, thereby facilitating wound healing (Heo, 2022). This process is associated with heightened biological effects related to wound healing and increased ex-pression of anti-inflammatory molecules. The aforementioned studies indicate that the development of an engineered exosome capable of loading functional molecules holds promise in expediting wound repair through the remodeling of the wound microenvironment (Wu et al., 2019). However, it is important to note that the concept of the wound microenvironment is still evolving, and a comprehensive understanding of its intricate complexity and dynamic alterations necessitates further in-depth research. Moreover, there is a pressing need for more precise therapeutic targets in the context of trauma, as well as addressing the associated challenges.

#### 3.3.4 Improvement of complications

Severe wound infections or burn wounds not only present challenges in wound healing but also give rise to systemic inflammatory effects that can result in severe multi-organ dysfunction and even mortality (Shpichka et al., 2019). Localized wound treatment often fails to yield satisfactory outcomes, while systemic treatment alone lacks precision. However, through targeted modification of engineered exosomes, precise treatment can be achieved by specifically targeting damaged organs (Yang et al., 2023c).

Heart failure and acute kidney injury are grave complications arising from severe wound infections. When the body’s ability to regulate wound infection is compromised, it can progress to sepsis and eventually lead to multiorgan failure (Jnana et al., 2020). In the state of systemic inflammation, various organs, including the heart, kidneys, and certain tissues, experience varying degrees of hypoxia (Raghav et al., 2021). Consequently, it becomes crucial to administer organ supportive and protective therapy. Mentkowski and Lang (2019) devised an engineered exosome that expressed a cardiomyocyte-specific binding peptide, enhancing its retention in the heart by facilitating targeted binding to cardiomyocytes (Liu et al., 2022b). Furthermore, Hu et al. (2022) discovered that exosomes were predominantly concentrated at the site of injured kidneys and effectively mitigated renal injury within 1 day of administration (Enescu et al., 2022). To enhance the accumulation of exosomes in the damaged kidney, Tang et al. (2021) developed an engineered exosome incorporating the targeting peptide KIM-1 (132). This modified exosome can selectively accumulate in the injured renal tubules, potentially ameliorating kidney injury.

Additionally, comprehensive analysis of the wound flora, accurate diagnosis, and effective infection treatment are crucial in managing severe wound infections. Identifying the predominant flora responsible for wound infections and developing targeted antimicrobial therapies are pivotal in promoting wound healing (Hu et al., 2022). Alok Raghav et al. proposed the engineering of exosomes loaded with biologically active molecules and antibiotic-like nonbiologically active substances, followed by targeted modification to confer specificity towards the bacterial population causing the wound (Raghav et al., 2021). These engineered exosomes can selectively deliver their regenerative and antimicrobial proper-ties to wound-specific bacteria, thus opening up new avenues for the treatment of severely infected wounds.

The targeting design of engineered exosomes can increase the aggregation of engineered exosomes at the damaged site to achieve high efficiency and precision. For infections involving systemic inflammation, the combined application of multiple approaches provides a new strategy for the treatment of complications associated with traumatic infections.

## 4 Engineered exosomes combined with biomaterials

Although numerous studies have focused on the therapeutic potential of exosomes for trauma treatment, their clinical translation remains challenging due to issues with low yield and efficiency of application, as well as challenges related to exosome delivery and duration of drug action (Kimiz-Gebologlu and Oncel, 2022). In addition to the engineering of exosomes to enhance their functional properties, it is imperative to consider the appropriate and effective application of exosomes to wound sites in order to achieve more efficient therapeutic outcomes (Pan et al., 2022). Some re-searchers have proposed combining engineered exosomes with various biomaterials for this purpose. In this context, biomaterials serve as carriers that effectively promote tissue repair by preserving exosome activity, prolonging exosome action, and facilitating slow exosome release.

Biomaterials comprise natural and synthetic elements. The biodegradable nature of natural biomaterials renders them advantageous for their biocompatibility in the body. Natural biomaterials, such as chitosan, fibrin, and hyaluronic acid, recreate the extracellular matrix environment, sustaining cellular activity for the efficient exchange of exosomes and cells to promote a synergistic repair effect (Lv et al., 2021; Prasathkumar and Sadhasivam, 2021). Synthetic biomaterials are primarily designed to be more flexible, improving the traumatic inflammatory microenvironment (Kalelkar et al., 2022), and facilitating loaded exosomes’ communication with cells in a favorable microenvironment for signaling (Fu, 2021; Nii and Katayama, 2021). Therefore, engineering exosomes loaded with functional therapeutics and targeting biomaterials may increase the binding stability of exosomes to biomaterials and enhance their biological function.

On the basis of the above analysis, the combination of functional drug-loaded exosomes and biomaterial targeting may increase the stability of exosome binding and thus the biological function of the exosome. Therefore, understanding biomaterial properties can lead to the development of a new strategy using engineered exosomes to bind biomaterials, resulting in a novel bioactive dressing to treat wounds.

For instance, VH298 is a hypoxia-inducible factor-1 alpha (HIF-1α) stabilizing agent, and overexpression of VH298 increases the levels of HIF-1α, which leads to enhanced angiogenesis and promotes wound healing in hypoxic environments. VH298-loaded exosomes were designed using a co-incubation method and subsequently combined with gelatin methacrylate (GelMA) hydrogel, GelMA was reported to store exosomes for long periods of time (Wang et al., 2022c). This composite system achieved sustained release and uniform distribution of exosomes on the wound site. By doing so, it enabled stable exogenous activation of the HIF-1α pathway, promoting angiogenesis and wound healing.

Furthermore, miR146a has been discovered to function as an anti-inflammatory modulator, Li et al. (2023a) developed engineered exosomes loaded with miR146a and created a protein-binding peptide using phage technology, enabling the fusion of the engineered exosome surface protein with the filamentous patch protein. This approach improved the binding efficiency and stability of the engineered exosomes to biological materials. As a result, it effectively suppressed inflammation, stimulated angiogenesis, and accelerated wound healing.

This finding was the result of an analysis of previous research (Table 2). These studies clearly demonstrate that the application of engineered exosomes in conjunction with biomaterials can yield more pronounced therapeutic effects. This therapeutic strategy not only opens up new avenues for disease treatment but also holds tremendous potential for the development of related biomedical materials.

**TABLE 2:** Summary of research investigating engineered exosomes combined with biomaterials involvement in wound healing.

Cargo	Exo-souces	Engineering methodology	Function	Wound type	Biomaterial	Properties of biomaterials	References
VH298	Epidermal Stem Cells (ESCs)	Co-incubation	Promote angiogenesis; HIF-1α pathway	Diabetic wounds	Gelatin Methacryloyl (GelMA) hydrogel	Long-term storage of exosomes	Wang et al. (2022c)
Hypoxia and TNF-a	MSC	Preconditioning	Anti-inflammatory M2 polarizing and stabilizing hypoxia-inducible factor-1α (HIF-1α)	Diabetic wounds	Nano materials	Antibacterial activity	Sun et al. (2022)
CBDs, TKKTLR/CP05 (CRHSQMTVTSRL)	Umbilical cord mesenchymal stem cells (ucMSCs)	Fusion peptide technology	Target to collagen in biomaterials	Diabetic wounds	catecholamine chemistry was used to modify small intestinal submucosa (SIS)	Rich in collagen and various biologically active factors	Ma et al. (2022b)
—	Adipose Stem-Cell (ADSC)	—	Promote angiogenesis and Diabetic wound healing	Diabetic wounds	Adhesive thermosensitive multifunctional polysaccharide-based dressing (FEP)	efficient antibacterial activity/multidrug-resistant bacteria, Fast hemostatic ability, Self-healing behavior, and Tissue-adhesive and Good UV-shielding performance	Wang et al. (2019b)

## 5 Future perspectives

Studies have substantiated the ability of engineered exosomes with specific functions to precisely and effectively promote wound healing by activating distinct signaling pathways, offering a promising avenue for clinical wound repair. Nonetheless, the underlying mechanisms through which engineered exosomes facilitate wound healing remain incompletely understood, and the existing methods for isolating these exosomes still exhibit several limitations and challenges.

First, it is crucial to carefully evaluate the therapeutic application objectives, microenvironmental conditions, and drug administration in the design process, particularly when faced with complex trauma microenvironments. Exploring the combination of multiple engineered modifications and maximizing the active ingredients of engineered exosomes represents a promising strategy for in-depth investigation. Second, accurate quantification of key molecules or proteins essential for treatment is necessary when performing exosome modification. Third, despite the availability of various methods for preparing engineered exosomes, challenges persist in terms of the purity, quality, and yield of the prepared exosomes, as well as the storage conditions of loaded engineered exosomes, all of which require further refinement. Fourth, the degree to which the properties of modified exosomes change, either during the engineering process or in a therapeutic context, remains incompletely understood. This is primarily due to the demanding nature of exosome preparation for clinical translational applications, where the quality control of engineered exosomes is more stringent compared to naturally secreted exosomes. It is important to note that the mechanisms underlying skin regeneration and rejuvenation are highly complex and cannot be attributed to a single molecule or signaling pathway.

## 6 Conclusion

In conclusion, engineered exosomes play a crucial role in wound repair and hold promise as important drug carriers. In future studies, it will be essential to investigate the underlying mechanisms by exploring the full spectrum of signaling networks in detail and by gaining a better understanding of the biological functions of various exosome components. We must strive to identify exosomes that contain specific active components involved in the wound healing process and elucidate how these components promote tissue regeneration. By specifically modifying exosomes, we posit that engineered exosomes could provide targeted and tissue-specific benefits in trauma re-pair, thereby improving their therapeutic efficacy and reducing potential side effects.
